# Unobtrusive Bed Monitor State of the Art

**DOI:** 10.3390/s25061879

**Published:** 2025-03-18

**Authors:** Toshiyo Tamura, Ming Huang

**Affiliations:** 1 Future Robotics Organization, Waseda University, Tokyo 162-0044, Japan; 2 Shenzhen Institute of Advanced Technology, Chinese Academy of Sciences, Shenzhen 518055, China; 3 Graduate School of Information Science, Nara Institute of Science and Technology, Ikoma 630-0192, Japan; alex-mhuang@is.naist.jp

**Keywords:** unobtrusive bed monitor, ECG, BCG, sleep assessment, body movement

## Abstract

On average, people spend more than a quarter of their day in bed. If physiological information could be collected automatically while we sleep, it would be effective not only for health management but also for disease prevention. Unobtrusive bed monitoring devices have been developed over the past 30 years or so to detect physiological information without awareness, and this method attracted attention again in the 2020s, with the proliferation of deep learning, AI, and IoT. This section describes the current state of the art.

## 1. Introduction

The advancement in unobtrusive monitoring technologies has significantly transformed the field of physiological sensing, particularly in sleep and health monitoring. Traditional physiological measurements, such as electrocardiograms (ECGs) and polysomnography (PSG), require direct contact with the body, often involving adhesive electrodes, cumbersome wires, or wearable devices, which can cause discomfort and disrupt natural sleep patterns. To address these limitations, researchers have explored embedding sensing devices directly into beds, offering a non-intrusive, automated method for continuous physiological monitoring.

Embedded bed sensors leverage various principles, including force, motion, pressure, bioelectric signals, and temperature detection, to capture vital physiological parameters without direct skin contact. The primary goal of these systems is to extract meaningful health-related information—such as heart rate, respiratory patterns, body movement, and sleep quality—while ensuring user comfort and minimizing behavioral interference. Several sensor types have been investigated, ranging from load cells and radar sensors to capacitive ECG and ballistocardiography (BCG), each with unique advantages and challenges in capturing physiological signals.

This review systematically examines the development and applications of bed-embedded sensors for physiological monitoring. Section 2 provides an overview of the various sensing technologies embedded in beds, including force and motion sensors, pressure sensors, temperature arrays, bioelectrical signal acquisition methods, and emerging approaches such as video-based monitoring and gas sensors. Section 3 discusses the physiological parameters derived from these sensing methods, with a focus on sleep quality assessment, heart rate and respiratory rate estimation, and their potential role in diagnosing sleep disorders. Section 4 explores the integration of these monitoring systems in clinical trials and real-world applications, including intensive care settings, nursing homes, and sleep disorder diagnostics. Finally, Section 5 presents future perspectives, highlighting the challenges, limitations, and opportunities for advancing unobtrusive bed monitoring technologies.

By compiling and analyzing recent advancements in bed-embedded physiological sensing, this review aims to provide insights into the evolving landscape of non-contact health monitoring, paving the way for future innovations in personalized and continuous health assessment.

## 2. Sensing Devices Embedded in the Bed

The concept of unobtrusive bed monitoring is either to embed various sensors in the bed or take indirect measurements by using bio-magnetic and optical sensors to monitor physiological parameters. The points of these measurements are indirectly obtained from physiological parameters such as force, motion, bioelectric signals, temperature, and so on. The various bed sensors have tried to monitor physiological conditions. This section presents the attempts at using sensors embedded in the bed.

### 2.1. History

The first attempt at an unobtrusive bed monitor was made in 1979 with the development of the static charge-sensitive bed (SCSB), which unobtrusively detects body movement [1]. Body movement detection allows for the prediction of sleep stages, heart rate, and respiratory rate. Later, literature was published on bed scales using load cells [2], and the patent for such devices appeared in 2007 [3].

In the 1990s, the development of miniature sensors and advances in high-speed data transmission with the advent of the Internet led to proposals for unobtrusive measurement techniques such as those used in smart houses. Togawa [4] proposed collecting physiological information by attaching sensors to furniture, which went beyond the scope of conventional measurement techniques.

The initial sensor selection criteria were based on the sensors used for physiological measurements with safety and viability, for example, a load cell for weight measurement and a thermistor for thermometry. For electrocardiograms, electroconductive fibers were used in the early stage. To avoid noise and uncertain signals, capacitive electrocardiogram electrodes were developed. This development was a new attempt for unobtrusive devices. Attempts were also made to measure information from weight changes with a ballistocardiogram. In addition, many other existing technologies, such as video imaging, μ wave, and mm-wave, were applied to monitor physiological information.

The following sections will discuss the bed-embedded and the indirect optical and electromagnetic wave measurements separately.

### 2.2. Bed-Embedded Sensors

#### 2.2.1. Load Cells for Force and Motion Detection

Force measurements can be obtained from load cells, which are available in several types: hydraulic, pneumatic, strain gauge, and capacitive. The simplest way is to install load cell sensors on the four legs of the bed. The load cell covers forces such as tension, compression, pressure, or torque into the electrical signals. The changes in resistance represent the changes in weight. The most common structure uses four force strain gauges in a bridge configuration, where changes in resistance are measured.

**Figure 1:**
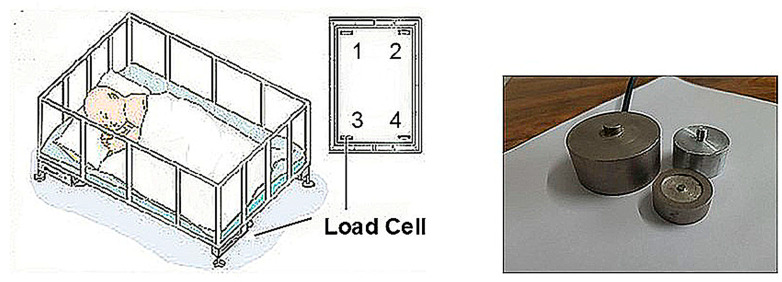
(**Left**) Physiological signal monitoring bed based on load cells [5]; (**Right**) load cell sensors.

Since the resistance change of a strain gauge is minimal, it is typically mounted in a Wheatstone bridge circuit. Four load cells are installed onto the four corners of the bed to provide accurate measurements. The accuracy of force measurement should be that used in body scales with an error of 100 g or less. Body movement can be detected by balancing the load cells at the four corners, as shown in Figure 1 [5]. Motion detection is achieved by estimating the energy in each load cell signal over short segments to capture the variations caused by movement. The most commonly used load cell types for hospital beds include planar, bending beam, and shear beam. Shear beam and bending beam cells are typically installed between each castor wheel and its attachment point beneath the bed. The output from each load cell is collected and summed to provide an overall measurement [2,6,7].

Load cells are widely used in various applications, including body weight and force measurement in beds, with commercial systems available. However, their use in medical settings faces challenges with motion artifacts, particularly when patients move, which can distort measurements. While the technology offers high precision in detecting body shifts and pressure changes, its primary drawback is sensitivity to external factors like vibrations or weight changes, which can interfere with accuracy. Additionally, installing and calibrating multiple load cells can be costly and complex, limiting their widespread adoption. Despite these challenges, load cells remain valuable for detecting subtle body movements and are critical for preventing conditions like pressure sores.

#### 2.2.2. Pressure Sensors

This method uses the air pressure of a mattress as a measure of changes in weight and/or body posture changes [8,9,10].

**Figure 2:**
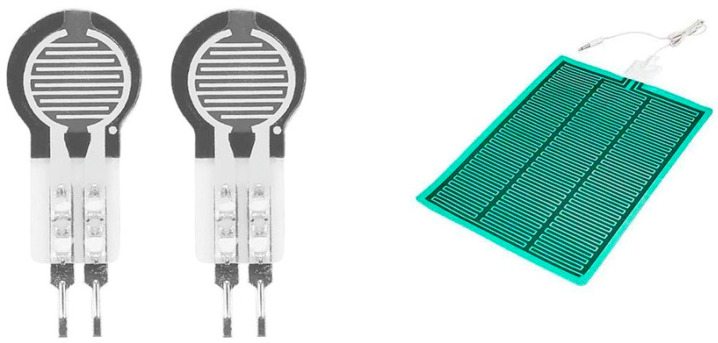
Thin-film pressure sensor (**Left**) and array (**Right**).

The pressure sensor is also embedded in the bed. Figure 2 shows examples of pressure sensors made of thin, flexible materials. The body movement and pressure changes are monitored in several ways. The pressure sensor is installed into the air mattress and measures the weight changes. The sensors can measure small pressure changes induced by the heartbeat. The captured signal requires advanced processing such as applying filters and amplifiers before the analog signal is ready for the next analysis. The peak signal is detected and the heart rate can be calculated.

**Figure 3:**
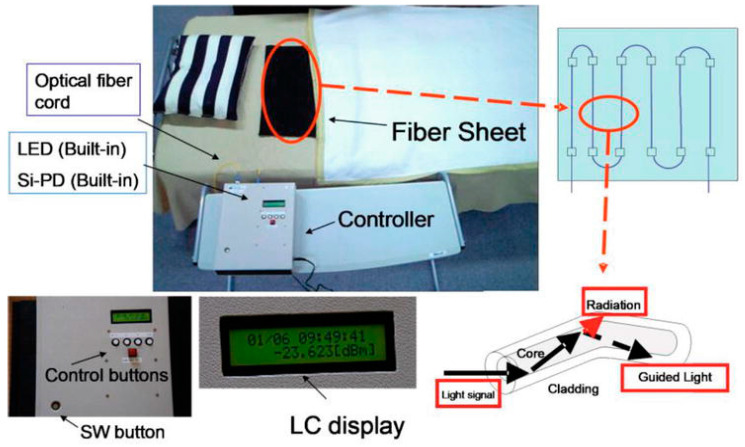
Fiber-optic pressure sensor system [11].

Optical fiber is also a candidate for monitoring pressure changes. A speckle output from multimodal optical fiber gives pressure changes and the fiber array can reveal the posture changes [11,12]. An example of an experimental system is shown in Figure 3 [11].

A fiber Bragg grating (FBG) is a type of refractive index modulation (diffraction grating) formed in the core of an optical fiber by irradiation with laser light. When incident light passes through the FBG, only light at the Bragg wavelength is reflected by the gating (reflected light), while light at other wavelengths passes through intact (referred to as transmitted light). If an external force is applied to the FBG, the optical fiber undergoes deformation, altering the spacing of the diffraction grating and, consequently, the Bragg wavelength. The basic mechanism of the FBG sensor is to detect this change in Bragg wavelength, known as wavelength shift, and thereby measure the external force [12,13].

Pressure sensors have been implemented in some medical bed systems, particularly for pressure ulcer prevention, but their use is still limited by issues with sensitivity and signal distortion from external factors like bed coverings or patient movement. The key advantage is their ability to detect body movements, heart rate, and respiration in a non-invasive, continuous manner, offering insights into changes in health status. However, their performance can be compromised by environmental variables such as temperature or humidity, which may lead to inaccurate readings. Furthermore, their resolution can be insufficient to detect minor body shifts, limiting their utility in certain medical applications. Ongoing research is focused on improving sensor sensitivity and integration with other monitoring systems.

#### 2.2.3. Temperature Sensors and Array

**Figure 4:**
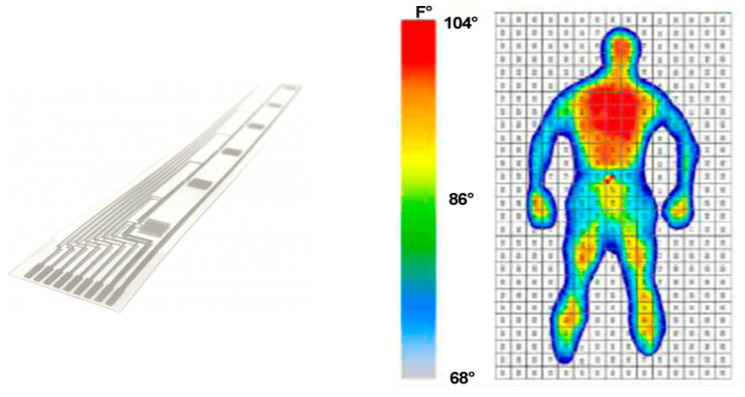
(**Left**) Thermex^®^ Tactile Surface Temperature; (**Right**) matrix arrangement sensor.

Temperature sensors such as thermistors and IC temperature sensors are primarily used to measure the bed temperature. Thermistors exhibit a non-linear relationship between electrical resistance and temperature, which is linearized using a bridge circuit. In contrast, IC sensors provide a unique temperature reading. Simple sensor arrays and metrics types have been tried [14,15,16]. For body temperature measurement, an accuracy of 0.3 °C is acceptable. Currently, flexible sensor arrays and matrix sensors are available on the market, as shown in Figure 4. These sensors are attached to the bed sheet. A temperature distribution of multi-channel thermistors or IC sensors is calculated. The temperature profile or time course of temperature change is detected, and then the sleep quality can be evaluated. The time of getting out of bed can also be determined. However, the distal skin temperature estimated from the bed temperature has an accuracy of approximately ±1 °C, which is insufficient for accurately estimating skin temperature [17].

Temperature sensors embedded in beds are still in the developmental stage for widespread health monitoring, focusing more on ambient temperature rather than direct body temperature. While some commercial systems exist, challenges with accuracy, particularly in differentiating between body and ambient temperature, remain. These sensors are advantageous for continuous, non-invasive body temperature monitoring, which is crucial for detecting infections or changes in health status. However, they can be affected by environmental factors such as humidity, bed coverings, or sweat, which can interfere with their accuracy. The technology needs further refinement to improve the accuracy of skin temperature measurements, especially in dynamic environments.

#### 2.2.4. Electric Signals

(a) ECG

**Figure 5:**
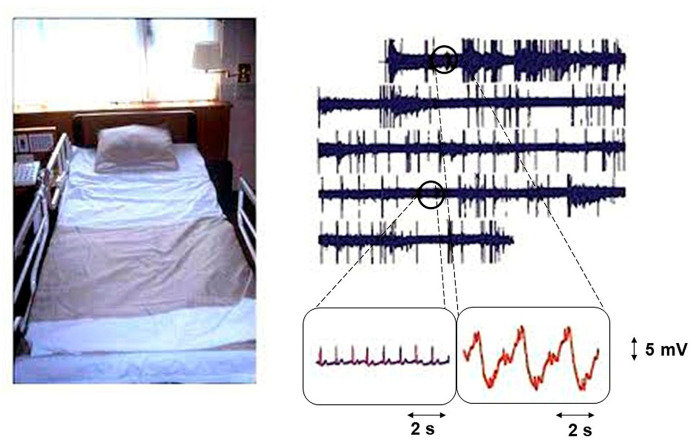
Bed ECG monitor (**Left**) and ECG signal (**right**) obtained during sleep [18].

Electrical signals are usually acquired through electrodes attached to the surface of the skin. For unobtrusive recording, the electrodes are attached to the surface of the bed. The simplest way to obtain ECG signals is by using an electroconductive sheet (e-textile electrode). Textiles can be rendered electrically conductive by integrating metals, carbon materials, or conductive polymers into the textile structure (fibers, yarns, or fabrics) at different stages using various techniques. In this way, devices that monitor physiological activities such as heart activities or electrocardiographic (ECG) signals do not require direct conductive contact between the device and bare skin. This method uses an array of high-impedance active electrodes attached to the mattress and an indirect skin contact ground consisting of a large conductive textile sheet. The signal quality is lower, and the motion artifacts are greater, than with conventional direct contact measurements. Figure 5 illustrates ECG monitoring with the textile sensor at night [18]. Motion artifacts affect the ECG signals, and when the user moves, the ECG signal may not be detected. ECG signals are reliably detected only in a resting state (Figure 5, bottom right).

The quality of the signal obtained from the bed sheet electrodes is sufficient for computer-assisted arrhythmia detection. The method can be used for HRV assessment with easy discrimination of R-peaks [19,20]. Despite the poor signal quality, it has been reported that sleep stage classification with 78% accuracy is possible based on HRV estimation [19].

(b) Capacitive ECG

Non-contact capacitive measurement using capacitive grounding has been developed and applied to a non-intrusive ECG measurement in daily life. Many ongoing studies are investigating how to improve the quality and ease of applying electrodes, and thus, extending their applications in ubiquitous healthcare from attached-on-object measurements to non-contact ECG measurements to improve the signal quality [21]. The small electric current generated by the heartbeat is recorded. It can detect arrhythmia due to an irregular pulse, enlargement or hypertrophy of the heart, and ischemic heart disease such as angina pectoris and myocardial infarction.

Capacitive ECG (cECG) is a non-contact method of detecting and recording cardiac impulses that uses capacitance instead of the traditional conductive method. It is a potential alternative to traditional contact-based ECG technology and has many advantages, including the following.

**Figure 6:**
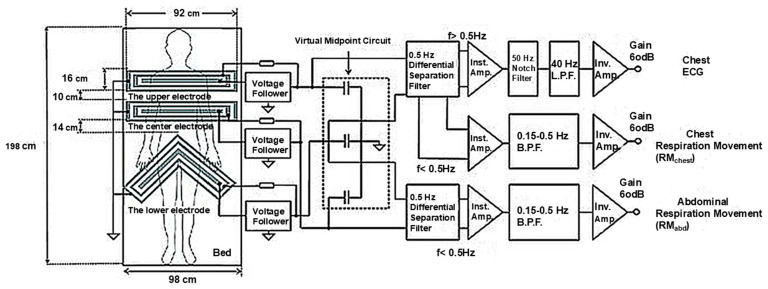
A typical example of ECG monitoring with capacitive electrodes [22].

cECG can be achieved by capacitively coupling biopotential amplifiers to the patient. Figure 6 shows a typical example of an electrical circuit [22]. Typical source capacitances can be on the order of 1–100 pF for frequencies of interest (0.05–150 Hz). Maintaining constant signal amplification at such low frequencies is the major challenge in the design of a cECG acquisition system. The feasibility of such amplifiers has been demonstrated, and non-linear bias networks can provide a method for transient suppression [22]. In addition, the ability of capacitive electrodes to acquire a signal through clothing and the potential to integrate these electrodes in an array opens up many possibilities.

cECG eliminates the need for trained professionals to perform the ECG acquisition on patients, as it can be performed through clothing and on unprepared skin. cECG acquisition through clothing does not cause skin irritation and therefore has no application time limit, thus facilitating long-term, continuous monitoring [23,24,25,26,27].

cECG is an emerging technology still in the research phase, offering a non-contact method for heart rate and respiratory monitoring. It has the advantage of being unobtrusive, improving patient comfort by eliminating the need for direct contact with the skin. However, it is prone to electromagnetic interference and signal degradation in noisy environments, which can limit its application in clinical settings. Additionally, capacitive ECG has lower accuracy compared with traditional ECG systems, especially when the patient moves, making it less reliable for precise health monitoring.

(c) BCG

**Figure 7:**
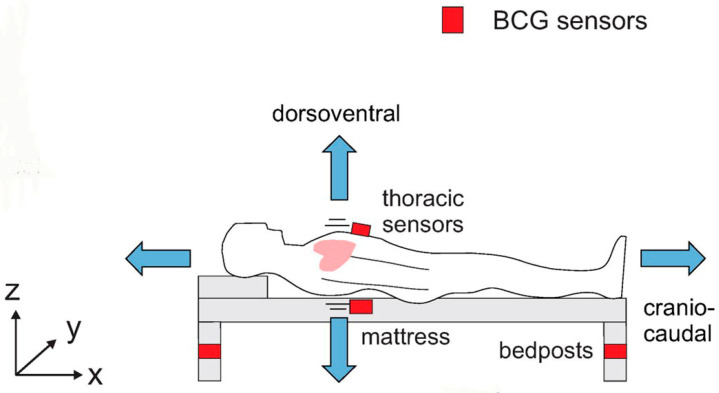
BCG monitoring system with embedded accelerometers or load cells (modified from Figure 5 in [30]).

A ballistocardiogram (BCG) is a technique that measures the heartbeat by detecting small body movements caused by the contraction of the heart. Unlike electrocardiograms, which capture the electrical movement of the heart, the BCG detects the body movements that accompany the physical movement of the heart. The bed sensor uses an ultra-sensitive accelerometer to capture these minute vibrations (Figure 7). In addition, algorithms embedded in the microcontroller extract biological signals such as pulse rate. By combining the biological signals extracted by these products in the system, various biological signals and bed conditions can be detected, including pulse rate, respiratory rate, heart rate variability (correlated with stress level), cardiac output, bed status detection (presence, absence, or movement of a person in bed), and so on [28,29,30,31].

**Figure 8:**
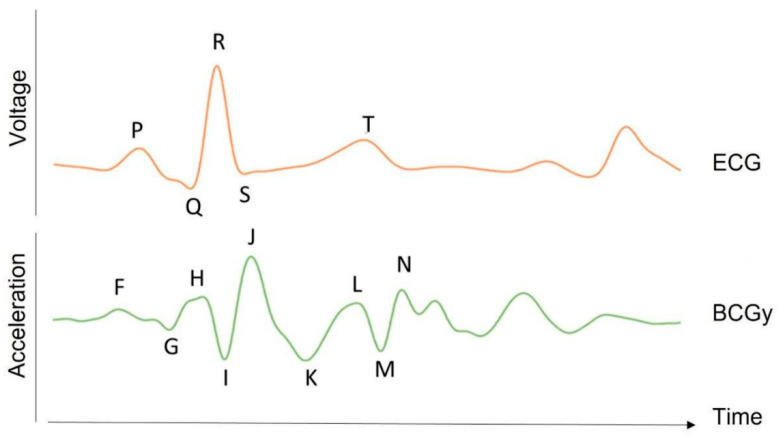
BCG waveform. The time traces of the BCG acceleration signal in the longitudinal axis (y) for a healthy subject are shown together with the ECG signal in one heartbeat (in arbitrary units). BCG waves show the “F” to “N” waves, which are typical of BCG recordings [31].

The BCG uses accelerometers mounted on the bed to detect the slight body vibrations caused by the heartbeat. Because these vibrations of the heart are extremely subtle, sensors that are about 50 times more accurate than typical accelerometers are used. Figure 8 shows the typical BCD waveform with ECG signal.

BCG sensors such as polyvinylidene fluoride film-based sensors, electromechanical films, strain gauges, hydraulic sensors, micro-bend fiber-optic sensors, and fiber Bragg grating sensors have been integrated into furniture such as mattresses, pillows, chairs, beds, or even weighing scales, to capture BCG signals and thereby measure vital signs.

BCG is still in the early stages of development. BCG sensors can detect subtle body vibrations caused by cardiac activity, offering the advantage of continuous monitoring without direct contact. However, motion artifacts from patient movement remain a significant challenge, degrading signal quality. The technology’s accuracy is lower compared with more established methods like ECG, and further research is needed to enhance its reliability for medical applications.

(d) Static Charge-Sensitive Bed (SCSB)

**Figure 9:**
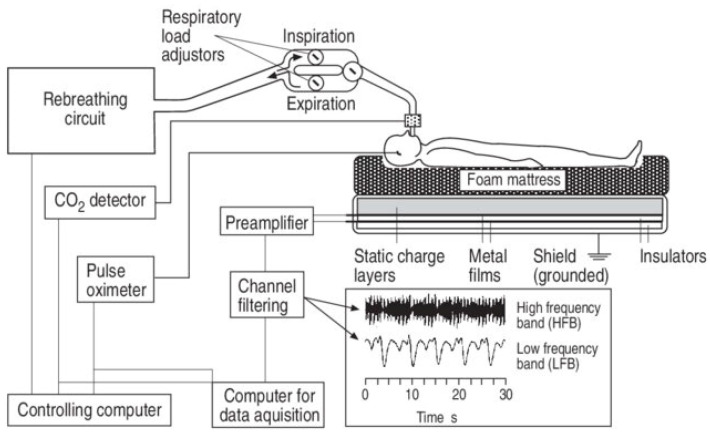
The recording setup with the static charge-sensitive bed (SCSB) [36].

An electrostatic charge is generated whenever two surfaces are in relative motion. When two surfaces rub together, electrons from the surface atoms of each material come close together, creating a static charge. The SCSB detects these static charges to record body movement and other physiological signals. The SCSB is capable of recording the ballistocardiogram (BCG) and respiratory movement simultaneously by selectively filtering the original signal [1,32,33,34,35,36]. Figure 9 shows an example of a recording system with a static charge-sensitive bed [36].

A material similar to the Emfit sensor is also applied to the bed monitor. The Emfit sensor is based on a permanent electric charge inside the cellular structure of the sensor core. With a change in thickness, the Emfit sensor generates a corresponding charge and voltage to appear on the electrodes [37]. This principle is analogous to that of the static charge-sensitive bed (SCSB), which also relies on the detection of static charges to monitor body movement and physiological signals.

SCSB technology is a very old technology, offering the potential for continuous non-contact monitoring of physiological signals. The advantage of the SCSB is its ability to detect body movements and physiological signals without direct contact, which improves patient comfort. However, it struggles with low sensitivity to small movements and is highly susceptible to signal noise, especially from external sources like bed materials or vibrations. The complexity of signal processing algorithms required to filter out noise is a significant disadvantage.

#### 2.2.5. Gas Sensor

There have been attempts to place gas sensors in beds for the early detection of incontinence and defecation. The main odor emitted from urine is NH_4_, and the main odor emitted from feces is H_2_S, both of which could be detected by the gas sensors. The design and implementation of a portable sheet has been developed for real-time monitoring of the odor of excrement for the health of infants and the totally bedridden elderly [38,39,40,41].

### 2.3. Indirect Sensing by Optical and Biomagnetic Sensors

#### 2.3.1. Video and Camera Images

Techniques have also been proposed to determine body movement and the respiratory breathing rate from different images of video frames and optical flow. In image sequences, analyzing how the image changes over time provides valuable information about the scene. The motion of points in the image, as described by the optical flow field, is an important cue in many computer vision tasks, such as tracking, motion segmentation, and structure-from-motion. It is common to estimate a dense optical flow field that provides a displacement vector for each pixel in the first image, describing where that pixel moved to in the second image [42,43]. The multisensory information from the multispectral video camera consists of RGB, NIR, and FIR, to increase the accuracy and robustness of the signals [44].

**Figure 10:**
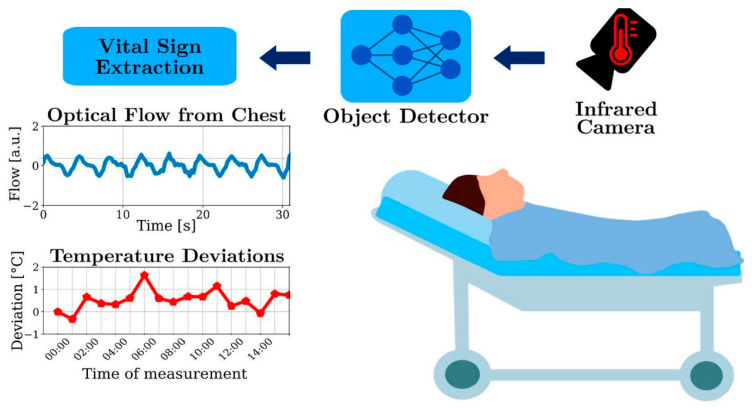
Camera-based temperature distribution monitor [45].

The temperature distribution can be obtained by an infrared camera as shown in Figure 10 [45].

Optical and video-based sensors are still in the early stages of development for health monitoring, with some systems able to detect body movement and respiration. These sensors offer the advantage of being non-invasive and capable of monitoring patients continuously without requiring physical contact. However, they are highly sensitive to environmental conditions like lighting and obstructions, which can significantly impact their effectiveness, and they also raise privacy concerns. The technology also faces challenges in extracting useful data from video images due to the complexity of human movement and health conditions, making it less reliable for precise health assessments at this stage. Further advancements are needed to improve signal processing and enhance system robustness in varied environments.

#### 2.3.2. Radar Sensor

The measurement using radio waves does not generally require contact with the body and does not disturb the user with various problems associated with wearing sensors [46,47,48]. In addition, the spatial resolution is not high enough to identify a human face, so there are fewer privacy concerns. In addition to radio waves, microwaves and millimeter waves can penetrate clothing and detect movements on the human body surface.

These sensors do not directly detect the movement of the lungs or heart but measure the minute skin movements associated with them. Although there are individual variations, it is generally accepted that the chest displacement caused by normal breathing in adults is on the order of a few millimeters, and the chest displacement caused by heartbeats is on the order of tens of microns. The phase of the data received by millimeter wave radar is proportional to the amount of minute skin displacement due to respiration and heartbeat divided by the radar wavelength; thus, the time variation of this phase is used to estimate vital information.

Obviously, data analysis is easier when the amount of phase variation with skin changes is greater. Radars have various bandwidths, and the phase fluctuations corresponding to distance fluctuations are larger in millimeter waves. For the high-frequency band, the target phase variation can be obtained with a higher distance variation, which is advantageous for detecting minute variations such as a heartbeat. Regarding the scattering of electromagnetic waves inside the human body, reflection from the skin surface is dominant in the high-frequency band, so the observed phase fluctuations can be detected as fluctuations on the skin surface.

**Figure 11:**
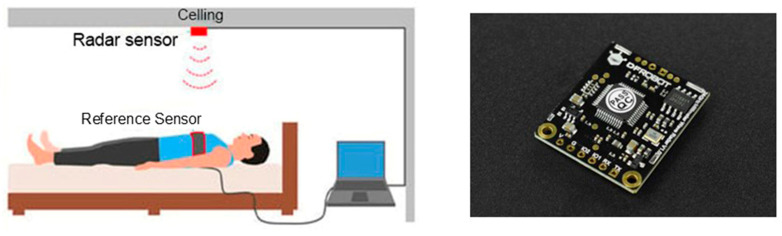
(**Left**) Radar installed in the room [48]; (**Right**) mm wave radar 24 FGHz Huan presence detection sensor.

The laser sensor, mounted directly above the bed, detects movement on the patient’s body surface as depicted in Figure 11 [48].

Radar sensors, particularly those based on microwave or millimeter-wave technologies, are becoming increasingly relevant for non-contact health monitoring, including in bed systems. The key advantage of radar sensors is their ability to monitor vital signs such as heart rate and respiration without requiring direct physical contact with the patient, making them ideal for long-term, continuous monitoring. However, radar sensors come with several disadvantages. They can be significantly affected by interference from other electronic devices or environmental factors, such as humidity or temperature fluctuations, which may degrade signal accuracy. Additionally, radar systems often require complex signal processing algorithms to distinguish between noise and meaningful physiological signals, and the data can sometimes be difficult to interpret without advanced processing. Despite these challenges, radar sensors are still in the experimental phase, but they are showing promise in both clinical and home health-monitoring applications, with ongoing research focused on improving their robustness and accuracy.

## 3. Physiological Parameters

From the sensors shown in Section 2, the information obtained includes heart rate, respiratory rate, HRV (a guide to stress), bed temperature variation, body movement, and body weight. This section presents in detail other possibilities of physiological parameters.

### 3.1. Sleep Quality and Body Movement

Body movement during sleep is known to correlate with sleep quality [49]. Excessive body movement is associated with poor sleep quality. A simple assessment involves observing body posture during sleep. Load-cell-type beds and bed mattresses were applied in [50,51]. Recorded heart rate and long-term heart rate variability (HRV), presented in Section 3.2, are sometimes used to assess sleep quality. The combination of heart rate and movement characteristics may be a suitable alternative for sleep staging, with the advantage of being low cost and simple.

For most of the sleep continuity variables (sleep latency, number of awakenings > 5 min, awakenings after sleep onset, and sleep efficiency), these measures serve as appropriate indicators of sleep quality across the lifespan [52].

Sleep quality can be precisely compared with the “gold standard” polysomnogram for objective sleep assessment. A polysomnogram (PSG) uses an electroencephalogram, electrooculogram, electromyogram, electrocardiogram, and pulse oximetry, as well as airflow and respiratory effort, to evaluate the sleep structure and the occurrence of sleep disorders. However, it is expensive, complicated, requires a strict measurement environment, and it cannot be widely used. If a bed monitor could be used as an alternative, the patients could avoid uncomfortable testing. However, the challenge lies in which combination of parameters detected by the bed monitor could provide sufficient information to approximate that from the PSG [53,54,55].

It is reported that the binary classification of slow-wave sleep versus non-slow-wave sleep achieves relatively high agreement between the bed monitor and PSG [53]. Siyahjani et al. [55] showed the evaluation of the performance in an unobtrusive bed monitor based on BCG. Epoch-by-epoch HR, BR, sleep vs. wake, mean overnight HR and BR, and summary sleep variables were compared. Epoch-by-epoch HR and BR were highly correlated with PSG, as were estimates of mean overnight HR and BR. The calculated agreement for sleep versus wake detection showed high sensitivity but poor specificity. For overnight summary variables, the unobtrusive bed monitor provided relatively accurate detection of sleep efficiency (SE), sleep onset latency (SOL), total sleep time (TST), and wake after sleep onset (WASO). Future work will build on these results by focusing on sleep stage detection using an unobtrusive bed platform. Overall, the unobtrusive bed monitor can provide reliable metrics to characterize human sleep unobtrusively in real-life conditions.

In summary, sleep quality can be detected and calculated using data obtained from mechanical sensors and fiber-optic sensors. Most of the literature discusses data in a laboratory environment, and a few studies are undergoing clinical trials. It is clear that the effectiveness of in-home sleep monitoring is lacking [10,56,57].

In recent years, sleep depth algorithms have been proposed through the mathematical analysis of signals obtained from surface ECGs and BCGs, and they have been applied to fiber-optic-based bed ECGs and BCGs to assess sleep. However, accuracy has not been achieved due to low signal intensity [58].

### 3.2. Heart Rate and Respiratory Rate

Heart rate and respiratory rate are the most popular parameters in bed monitors. The principle of measurement has long relied on the detection of R-waves from ECGs obtained from the electroconductive sheets to estimate heart rate and respiratory rate from the envelope of the ECGs [9,10,11,59,60]. Additionally, there are several methods for estimating heart rate and respiratory rate from load cells under the bed supports [2,5,6,61,62], bed mats with pressure sensors [10,46,47,48,63,64,65], static charge-sensitive beds [1,33,34,35,36,37], capacitive ECGs [19,20,21,22,23,24,66], and BCG signals [25,26,27,28,29,30], as well as optical flow from video images [42,43,44]. ECG and BCG [10,11,12,13,14,15,16,17,18,19,20,21,22,23,24,25,26,27,28,29,30,31,32,33,34,35,36,37,38,39,40,41,42,43,44,45,46,47,48,49,50,51,52,53,54,55,56,57,58,59,60,61,62,63,64,65,66,67,68] signals have low signal-to-noise ratios, but the R-wave in the ECG signal can still be detected, allowing heart rate estimation. There have also been attempts to calculate heart rate and respiratory rate from changes in the light intensity in optical fibers [69].

In summary, bed monitors can unobtrusively obtain heart rate, respiratory rate, and heart rate variability using several principles.

### 3.3. ECG and Ballistocardiogram

The signal from ECGs and ballistocardiograms is often insufficient for diagnosis because it is low and noisy. The BCG parameters (J-peak to J-peak interval, J-peak to K-peak amplitude, and the most significant frequency component) change slowly and are correlated with time, so motion detection and removal methods are useful. Autoregressive model-based tracking and Wiener smoother-based parameter estimation are effective for heart rate detection, but the morphological analysis has not yet been achieved [70,71].

In summary, the heart rate and respiratory rate are effectively detected in bed monitors by ECG, BCG, Doppler radar, optical image, or RGB cameras, but no diagnosis has been suggested [72,73,74,75,76].

### 3.4. Blood Pressure

BCG-based estimation of blood pressure (BP) was attempted using piezoelectric bed sensors. BCG measured by bed sensors provides a convenient and unobtrusive method for overnight monitoring of blood pressure (BP). A combination of morphological features, spectral features, and fractal dimensions of the BCG signal has been used to estimate blood pressure. The proposed system was evaluated for its effectiveness in hospitalized patients and healthy subjects [77]. Additionally, oxygen saturation was also attempted using video images, but the camera-derived estimates were less accurate in clinical settings [78].

### 3.5. Body Temperature Measurement

There have been several attempts to monitor bed surface temperatures and changes in temperature profile with body movement and sleeping state [14,15,16]. However, no systematic research has been carried out. Body core temperature can serve as an important early predictor of fever. As the body temperature rises, the skin surface temperature in the bed also rises. This hypothesis is supported by a preliminary study [79]. Skin surface temperature may be an important early predictive sign before the onset of fever.

### 3.6. Odor Sensing

Methods to detect urination and defecation in bed have also been suggested but remain unproven. In other words, a method of attaching a sheet with an odor sensor to the bed for detection has been proposed, but the accuracy of this method has not been evaluated or reported.

## 4. Clinical Trials

Numerous clinical trials have been conducted to explore the potential of unobtrusive bed monitoring, particularly focusing on heart rate, respiratory rate, and heart rate-based sleep stage classification. Other studies, which are also discussed in this section, have evaluated various applications of bed monitoring systems.

### 4.1. Physiological Information for Babies

A major advantage of unobtrusive bed monitoring is the non-contact measurement, eliminating the need for sensor attachments to the body. The first development was body weight measurement with embedded load sensors under the bed legs. Body weight is rather difficult to estimate due to various disturbances such as clothing changing, eating, and urination. Despite these challenges, unobtrusive monitoring in neonates has shown potential.

A non-contact vision-based infrared respiratory monitor has been used to measure respiratory movement in the lower trunk. The captured images of the trunk region are compared with impedance pneumography and capsule pneumography, demonstrating comparable results with a relatively high spectral purity index [80,81].

In another approach, conductive textile fabric and conductive silver ink electrodes embedded in a delivery room mattress provide high-quality, repeatable ECG recordings. This device could potentially be used to automatically record the HR of newborns after delivery in a safe, fast, and reliable manner without the need to attach additional sensors [82].

Another study shows that a load-cell-based physiological signal monitoring cot could accurately capture BCGs and respiratory signals in infants. This study showed that the BCG-based technology estimated HR and breathing rates with average errors of 2.55% and 2.66%, respectively. This device represents an essential step toward unobtrusive physiological measurements for infants [5].

### 4.2. Intensive Care and Critical Care

In intensive and critical care settings, unobtrusive bed monitoring offers an alternative approach to minimize the “spaghetti syndrome” caused by excessive sensor attachments on patients. The goal is to reduce the number of sensors directly attached to a patient while still obtaining reliable physiological data. However, there are limited studies in this field.

One example is a study on respiratory rate detection using video-based methods performed on both single and multiple patients. The result showed that a reliable respiratory rate could be obtained, demonstrating the potential of this approach in intensive care settings [83].

### 4.3. Assistive Care in Nursing Homes

In elderly facilities and nursing homes, the unobtrusive bed sensors offer a potential solution for physiological monitoring. A recent observational study in a nursing home demonstrated the use of body weight measurement for this purpose. The study monitored body weight loss in nursing home residents who are particularly vulnerable, those with severe cognitive and physical dysfunction, for 3 months. This study aimed to prevent delays in addressing gradual functional decline due to nutrition issues, which can lead to weight loss, malnutrition, and ultimately increase the risk of death [84].

### 4.4. Sleep Apnea

Obstructive Sleep Apnea (OSA) has become the most prevalent non-communicable disease. Objective sleep assessment relies on the monitoring and comprehensive analysis of one or more human signals using specific sleep monitoring devices to assess sleep structure and sleep status. As mentioned in the previous section, PSG remains a gold standard device but requires excessive sensor attachments to the body. Various objective sleep monitoring tools have been developed to quantify sleep quality more precisely, and even for disease diagnosis, using biological signals exhibited by different sleep phases. Unobtrusive bed sensors have mainly attempted to estimate sleep parameters from the respiratory rate and heart rate. These measurements are gathered from pressure sensors, respiratory microphones set up with mattresses and/or pillowcases, video images of the upper bed sheet, as well as ECG and BCG signals. However, successful clinical trials in this area are still lacking.

### 4.5. Commercial Devices

**Figure 12:**
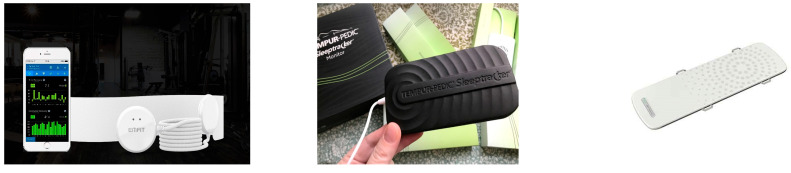
Sleep quality monitors: from the left, Emfit, Sleeptracker, and NemuriScan.

There are numerous sleep quality monitors available, but only a few are supported by substantial evidence. The devices that underwent validation studies are shown in this section. Table 1 shows the specifications of the commercial devices, and Figure 12 shows photographs of them.

The Emfit sensor [85] is an under-mattress BCG sensor that detects heartbeat, breathing, and other body movements. Its BCG signals are particularly useful for diagnosing Sleep Disorder Breathing (SDB) [37,86,87,88]. In the clinical rehabilitation setting, the device provides a solid foundation for the development of bed sensors tailored to address sleep types and sleep disorders, optimizing rehabilitation care [89].

The Sleeptracker-AI monitor [90] is a valid consumer-grade home sleep monitoring device designed for assessing sleep stage and sleep quality. It holds potential as a reliable tool for sleep health assessment and OSA screening in clinical settings [91].

The Nemuri SCAN (NSCAN) [92] incorporates a flexible, stretchable, and soft rubber-based tactile sensor sheet. An observational study attempted to measure the sleep in critically ill patients with this device. The comparison between the developed device and PSG showed moderate agreement, high sensitivity, and poor specificity in the ICU setting. A secondary comparison was conducted between objective parameters obtained from NSCAN and PSG as well as subjective data collected using the Richards–Campbell Sleep Questionnaire (RCSQ). The study revealed the need for further improvement to align subjective and objective sleep assessment [93].

The devices shown in Table 1 and Figure 12 mainly monitor patients’ sleep conditions, but the results were unsatisfactory. The reason for this was that the sensor moved with body motion during the measurements. Improving the geometry of the sensor is the best way.

In summary, the advantage of these devices is that you can take measurements without discomfort and without being aware that you are being measured. It is difficult to state the advantages and disadvantages of the measurement methods. For example, any method can be used to determine heart rate through sophisticated signal processing. However, there are problems with signal quality. We concluded that this indirect monitoring does not achieve concrete monitoring for physiological information.

## 5. Future Prospects

This review highlights discreet bed-monitoring devices and their developments. Initial attempts involved detecting body weight using load cells embedded in the bed legs. However, body weight detection was not consistently successful. Attempts were then made to measure the electrocardiogram using movement detection, the ballistocardiogram, and the electrocardiogram using capacitive electrodes. Unfortunately, the electronic circuit technology available during the early development phases was insufficient to capture accurate and noise-free data, leading to a temporary halt in research.

In the 2020s, advances in machine learning and deep learning brought this field back into the limelight, and a number of studies have been reported. In particular, there are many examples of AI being applied to sleep assessment. Sophisticated digital filtering and machine learning enable more accurate heart rate and arrhythmia detection. Further developments in signal processing and computational analysis have the potential to refine sleep stage and arrhythmia detection, paving the way for more effective and reliable devices.

Considering the aging society and the burden of care in elderly care facilities, bed monitors offer significant advantages. Detection of heart rate, respiratory rate, and heart rate variability in bedridden elderly patients is important for health management. In addition, the prevention of bed sores and control of weight by measuring the presence or absence of body movement are also potential applications. These systems can be used as screening and healthcare at home and in nursing homes. A design with high reliability and easy handling is needed.

## 6. Conclusions

Unobtrusive bed monitors have been comprehensively reviewed, encompassing various physiological parameters that can be detected and evaluated. While early studies failed to achieve satisfactory accuracy or validity, advances in hardware and the advances in machine learning and deep learning have rekindled research interest in this area. The concept of unobtrusive monitoring remains promising, with the expectation that instruments that provide clinically acceptable accuracy and validity will be developed in the near future.

## Figures and Tables

**Table 1:** Commercial devices with verification study.

Product Name	Emfit QS Sleep Tracker	Sleeptracker-AI^®^	NSCAN
Manufacturer	Emfit Ltd., Vaajakoski, Finland	Sleeptracker, Santa Cruz, CA, USA	Paramount Bed Co., Ltd., Tokyo, Japan
Principle	Ballistocardiography	Force	Pressure
Parameters	HR HRV Sleep period Sleep stage	BR Snoring HR, RR Sleep stage	HR, RR Sleep stage
Dimensions	542 mm L × 70 mm W × 1.4 mm THK	146.6 mm L × 77 mm W × 15 mm THK	245 mm L × 780 mm W × 15 mm THK
Sensor	Emfit‘s proprietary dynamic ferroelectret sensor	Piezoelectric sensors	A flexible, stretchable, and soft rubber-based tactile sensor sheet

## Data Availability

Data sharing is not applicable.
